# Reflections on project ECHO: qualitative findings from five different ECHO programs

**DOI:** 10.1080/10872981.2021.1936435

**Published:** 2021-06-02

**Authors:** Jon Agley, Janet Delong, Andrea Janota, Anyé Carson, Jeffrey Roberts, Gerardo Maupome

**Affiliations:** a Prevention Insights, Department of Applied Health Science, School of Public Health Bloomington, Indiana University Bloomington, Bloomington, Indiana, USA; b IUPUI ECHO Center & Center for Public Health Practice, Richard M. Fairbanks School of Public Health, Indiana University, Purdue University, Indianapolis (IUPUI), Indiana, USA; c Richard M. Fairbanks School of Public Health, Indiana University, Purdue University, Indianapolis (IUPUI), Indiana, USA

**Keywords:** ECHO, Project ECHO, medical education, continuing education, professional development

## Abstract

Project ECHO (Extension for Community Healthcare Outcomes) was developed in 2003 as an innovative model to facilitate continuing education and professional development. ECHO emphasizes ‘moving knowledge, not people.’ To accomplish this, ECHO programs use virtual collaboration and case-based learning to allow practitioners, including those in rural and underserved areas, to receive specialist training. The ECHO model has expanded rapidly and is now used in 44 countries. Preliminary research on ECHO’s efficacy and effectiveness has shown promising results, but evidence remains limited and appropriate research outcomes have not been clearly defined. To improve the evidence basis for ECHO, this study of 5 ECHO programs (cancer prevention/survivorship, integrated pain management, hepatitis C, HIV, and LGBTQ+ health care elucidated actionable insights about the ECHO programs and directions in which future evaluations and research might progress. This was a qualitative study following COREQ standards. A trained interviewer conducted 10 interviews and 5 focus groups with 25 unique, purposively sampled ECHO attendees (2 interviews and 1 focus group for each of the 5 programs). Data were transcribed verbatim and analyzed using the general inductive approach, then reviewed for reliability. We identified four major categories (reasons to join ECHO, value of participating in ECHO, ways to improve ECHO, and barriers to participation) composed of 23 primary codes. We suggest that thematic saturation was achieved, and a coherent narrative about ECHO emerged for discussion. Participants frequently indicated they received valuable learning experiences and thereby changed their practice; rigorous trials of learning and patient-level outcomes are warranted. This study also found support for the idea that the ECHO model should be studied for its role in convening communities of practice and reducing provider isolation as an outcome in itself. Additional implications, including for interprofessional education and model evolution, were also identified and discussed.

## Introduction

Project ECHO (Extension for Community Healthcare Outcomes) was developed at the University of New Mexico by what is now the ECHO Institute[1]. Though originally couched as a ‘disruptive innovation’ in medical education[2], ECHO is on the way to becoming ubiquitous. ECHO links specialists and other experts with non-specialist practitioners, including those in rural and underserved areas[3], using Zoom (Zoom Video Communications, Inc., San Jose, CA) or similar teleconferencing technology. The ECHO model is facilitated by ECHO Hubs, which are sites outside the ECHO Institute that utilize the model, must be approved and receive training from the Institute, and have to adhere to multiple procedural guidelines[4]. Hubs offer programs or tracks (vernacular differs by Hub) that provide telementoring and case-based learning to support continuing education, specialty training, and transfer of knowledge and skills needed to resolve complex clinical conditions[5]. A typical program might offer 60–90 minute sessions that include both a brief didactic learning presentation and collaborative review of 1 or more participant-submitted cases[6]. The case-based learning approach provides an exemplar of shared problem-solving and allows facilitators to ‘understand participants’ clinical roles and [call] on [them] to share their expertise and experiences.’[7]

Enthusiasm for the ECHO model has grown substantially; as of March 2021, there were 920 ECHO programs in 44 countries[8]. Evaluations of early program adoptions beyond the ECHO Institute suggested high levels of promise and implementation feasibility [9–11]. ECHO’s digital approach and standardized format were particularly well suited to the COVID-19 pandemic, during which the ECHO Institute was able to rapidly create 10 program tracks that included infectious diseases, critical care, and education[12].

To some extent, the evidence basis for ECHO is still catching up with the rapid proliferation of programs. In a 2016 systematic review of ECHO[13], most studies reported outcome data from levels 1 through 4 of Moore’s evaluation framework[14], often using surveys to examine participation levels, provider satisfaction, changes in knowledge, and competence. Some studies also captured objective knowledge (multiple choice questions) or used interviews to assess self-confidence. Unrelated to Moore’s framework, some studies also examined motivators and barriers to participating in ECHO[13]. A 2019 systematic review produced similar findings, which emphasized the importance of continuing to study ECHO[15]. Research conducted since the latter review has included a randomized trial of ECHO for caring for patients with autism, which reported mixed findings on learning outcomes[16], and a study suggesting that hepatitis C cure rates for non-specialists attending ECHO were not inferior to rates for specialists[17].

ECHO is a complex education innovation, so it is reasonable that studies have focused on disparate outcomes and topics and have used different approaches. In 2020, an expert panel identified challenges to building the evidence basis for Project ECHO and other ECHO-like models (EELM), including the need to ‘develop a clear understanding of EELM, what they are intended to accomplish, and the critical components of EELM that are necessary to meet their goals’ as well as ‘reporting on a broader set of EELM program characteristics.’[18]

Continued study of the ECHO model is warranted in multiple domains. While distal (patient-level) outcomes are important to study, research on ECHO qua medical education must clarify the outcomes that Hubs and participants hope to obtain from ECHO and the granular components that support programmatic efficacy. Multi-stage mixed methods approaches are likely to be useful at this stage; qualitative data can be used intentionally to develop and provide a framework for quantitative research[19]. Analyses of qualitative findings can then be used to inform the kinds of questions that can be asked, and the hypotheses that should be tested[20], as well as providing additional actionable information.

Thus far, the preponderance of qualitative ECHO studies has focused on single programs (e.g. pain management). However, our ECHO Hub used a uniform approach to collect data from five different ECHO projects in 2020 and early 2021. Those programs were offered by the IUPUI ECHO Center at the Richard M. Fairbanks School of Public Health at Indiana University-Purdue University, Indianapolis (IUPUI). Each of the programs had a different expert panel but shared key staff members, such as the project managers and the evaluator. The ECHO programs independently focused on cancer prevention/survivorship, integrated pain management, hepatitis C (HCV), human immunodeficiency virus (HIV), and health care for members of the LGBTQ+ community. By using a standardized approach to collect data from ECHO programs focused on disparate topics, the objective of this report was to elucidate both actionable insights about the ECHO programs as well as directions in which future evaluations and research might reasonably progress. In doing so, we investigated several broad questions: (1a) How do ECHO attendees perceive ECHO, including general, positive, and negative perceptions? (1b) How would ECHO attendees propose to change the program to correct perceived deficits? (2) Have ECHO attendees changed their professional practice since participating in ECHO, and if so, how? And finally, (3) How would an ‘ideal’ ECHO program appear and function?

## Methods

Study methods are reported according to the Consolidated Criteria for Reporting Qualitative Studies (COREQ)[21].

### Research team and reflexivity

Interviews and focus groups were conducted by JD (DPT, MSW, MHS, NBC-HWC), a training specialist and research associate at Prevention Insights in the Indiana University School of Public Health Bloomington. JD has extensive experience conducting interviews and focus groups and is also a member of the Motivational Interviewing Network of Trainers (MINT)[22]. JD did not have relationships with the participants/interviewees but was a participant in the ECHO program, and so was familiar to some respondents. They introduced themself and their role at the outset of each instance of data collection. They were selected to conduct data collection due to their experience with the ECHO program and conversational expertise through MINT.

### Study design

#### Participants and data collection

Participants were identified purposively by one author (AJ, Director of the IUPUI ECHO Center), separately by type of ECHO. Though purposive sampling typically is used to gather data from hard-to-reach populations[23], here the purpose was to ensure that invitees were sufficiently active in their ECHO program that they could provide in-depth feedback. Once participants had been identified, they were recruited to participate in either a focus group or an individual interview by another author (JD) using e-mail.

The decision to use both focus groups and individual interviews was made prior to the initiation of the study. This decision was both philosophical, as data gathered in the context of participant interaction conceptually differs from data gathered 1:1[24], and pragmatic, since a large, randomized comparison of the two approaches found that interviews may produce a wider range of categories but focus groups may elicit more sensitive themes[25]. As such, recruitment into interviews or focus groups was not differentiated and was based on scheduling and availability of participants.

A total of 15 data collection instances were completed (2 interviews and 1 small focus group for each of 5 ECHO programs). A total of 25 unique individuals provided data for the project, with an additional 3 seeming to agree to participate but not attending. Data collection occurred from May 2020 through February 2021; all recruitment was digital, and all conversations occurred over Zoom. Data collection was scheduled for each program to correspond with the end of the annual ‘program cycle.’

Topics and prompts used in the semi-structured guide were developed a priori by the study authors based on their experience with running ECHO programs since 2018. JD authored the first drafts, JA and AJ revised them, and then the team reviewed them several times for clarity and consistency (see Supplement 1). Partway through the project, several additional prompts were added to the guide to support quality improvement (noted in Supplement 1); however, qualitative review indicated that these points were typically addressed by participants without needing a prompt. The same guide was used for focus groups and interviews.

#### Theoretical assumptions

The research team used the general inductive approach [26] to develop categories and codes. In contrast to approaches using deductive analysis, which would focus on exploring predefined theories or frameworks, the general inductive approach emphasizes ‘allow[ing] findings to arise directly from analysis of raw data’ in a way that is ‘relevant to evaluation or research objectives.’[26] Procedurally, the approach included ‘preparation of raw data files,’ ‘close reading of the text,’ ‘creation of categories from multiple readings,’ and ‘continuing refinement.’[26] It also bore similarity to, but was not fully consistent with, inductive constant comparison analysis[27]. Finally, the modification of the question set partway through the study in response to emergent themes was consistent with grounded theory, but this was not a grounded theory analysis[28].

#### Transcription and saturation

All conversations were videorecorded (with permission) and were scheduled for 60 minutes, with some variability in actual length. All data were professionally transcribed by a vendor. Data saturation was not discussed at the outset of the project due to the nature of the original purpose of data collection. However, in inductive qualitative research, the standard for saturation typically is ‘theoretical saturation,’ meaning that analyzing additional data does not result in the identification of new categories or codes[29]. For this study, no additional categories or codes were identified while analyzing the final three transcripts out of 15. Thus, inductive thematic saturation might be inferred[29].

### Data analysis

All data from interviews and focus groups were coded in aggregate by JA. This was an inductive study focused on broad elucidation of ideas, so all relevant categories and codes that were identified were included in the emergent codebook. Iterative coding included a review for themes by TJ (three data sets) and JR (all data sets), and a final concordance review by AJ (three data sets). These secondary reviews included two individuals (TJ and JR) who were familiar with but not stakeholders in ECHO, and one individual (AJ) who was both a stakeholder and member of the ECHO program, providing triangulated assessment of the analytic credibility[26]. The final codebook consisted of four categories filled with 23 primary codes (see Table 1) that were developed during analysis. Consistent with the general inductive approach, instances of text were not required to belong exclusively to one code[30]. Rather, the codes and the categories were reflective of the overall concepts identified in the data that were collected. However, for clarity, specific statements that exemplify each of the codes within each category are provided in Table 2.

**Table 1.** Codebook and definitions

Designation	Name of Category/Code	Definition
**1.**	**Motivations to join ECHO**	**Things that caused participants to initially try ECHO or look into attending.**
1.a.	General continuing education	Anything having to do with wanting to build their own knowledge base, learn more, or grow as a professional.
1.b.	Help for rural or remote providers	Desire to access resources that might be hard to access from the participant’s current location or practice site.
1.c.	Intention to develop networking	Desire to build or grow a professional network by joining ECHO.
1.d.	Need for CE	ECHO was listed as a free CE opportunity.
**2.**	**Value of ECHO**	**Benefits or other positive outcomes people report from participating in ECHO (*as distinct from motivations to join initially*).**
2.a.	Networking	Networking with others (*as distinct from obtaining opinions from others*).
2.a.i.	Third-degree networking	Instances where networking extended beyond participants by at least one degree (e.g. descriptions of how people not attending ECHO were engaged in the ECHO network).
2.b.	Met a technical, legal, or CE requirement	Participant values the ability to meet a requirement by attending ECHO.
2.c.	Structure of ECHO	Something about the overall structure of the program, the norms of ECHO, or other unique features of ECHO was seen as a benefit.
2.d.	Information from didactics	Explicit mention of the value of didactic aspects of the ECHO program.
2.e.	Being able to present or address difficult cases	Value of the ability to either present or review others’ difficult cases.
2.f.	Interprofessional nature	The value of having different professions represented ‘at the table’ (whether in spokes, hubs, or both).
2.f.i.	Equity or lack of hierarchy	Some mentions of interprofessional experience also emphasized a lack of traditional hierarchy.
2.g.	Access to expert opinion	Value of being able to access expert opinion (*as distinct from being able to network with experts*).
2.h.	Changes to professional practice	Participant indicates that ECHO has changed their professional practice or has led to changes in their practice environment.
**3.**	**Ways ECHO can improve**	**Suggestions for how ECHO can be improved.**
3.a.	Facilitate networking	Participant comments related to additional ways in which ECHO might support networking.
3.b.	Structural changes	A sequence of ‘micro-themes’ related to specific modifications that could be made to details of the ECHO programs
3.b.i	Record didactic presentations	Comments about the importance of recording didactics (or, after this was implemented, the value of having them recorded).
3.b.ii	Modify didactic presentations	Suggestions related to how the didactic portion of ECHO could be improved.
3.b.iii	Allow more time for case management	Participant discussion that insufficient time is allocated to case management.
3.b.iv	Reconceptualize or minimize introductions	Critiques specific to the ECHO-based structured way in which participants introduce themselves.
3.c.	Expanding the ECHO community	Suggestions about ways to make the ECHO program ‘bigger’ or to extend beyond the tele-mentoring sessions.
3.d.	Consider braiding telemedicine with ECHO	Ideas about integrating the ECHO education model with clinical practice.
3.e.	Work to facilitate follow-up between sessions	Participant comments about improving continuity, both in terms of didactics (questions) and cases (‘what happened’).
3.f.	Miscellaneous ways to improve	One-off responses that were notable.
**4.**	**Barriers to participating**	**Things that made participating more difficult.**
4.a.	Time	Statements about the length of time for each session (e.g. duration).
4.b.	Scheduling	Statements about scheduling issues that are *independent of* the length of time.
4.c.	Miscellaneous barriers	One-off responses that were notable.

**Table 2.** Exemplar quotes

Designation	Name of Category/Code	Quote (*Source*)
**1.**	**Motivations to join ECHO**	
1.a.	General continuing education	‘There was another nurse practitioner doing this and then she was going to be retiring and so they were looking for somebody. I was a little hesitant, and so I started attending the ECHO before I agreed officially to do it … ’ (*HCV Focus Group*)
1.b.	Help for rural or remote providers	‘It’s really, truly worth it and I think we’re really lucky to have it because [Redacted] can be so rural in areas and having access to this can really help those rural providers too.’ (*HIV Interview 1*)
1.c.	Intention to develop networking	‘I’m working with care coordinators and providers from all different corners of the state, so it’s helpful to see what they’re dealing with in a rural setting, but also connect with them in other aspects.’ (*HIV Focus Group*)
1.d.	Need for CE	‘How I found ECHO is that I have to do these CHES credits. I don’t know why. When I was in graduate school, everybody was taking this CHES.’ (*HCV Interview 2*)
**2.**	**Value of ECHO**	
2.a.	Networking	‘But if my job is kind of expansive and I have to be a little bit of piece of everything then it’s really helpful. One, to have the information and, two, the networking opportunities. That’s been a big part of it as well.’ (*HIV Interview 2*)
2.a.i.	Third-degree networking	‘For me, it’s the tools and I’m able to share them with a vast, larger audience. Those tools are being used in other practices now. Even though the person wasn’t on the ECHO themselves, those tools are getting shared and spread even further.’ (*IPM Focus Group*)
2.b.	Met a technical, legal, or CE requirement	‘I also very much like the fact that for me, a pull is that it provides CHES credits and in a very easy, easy way.’ (*Cancer Interview 1*)
2.c.	Structure of ECHO	‘Also some of the ECHO principles where we all get to talk to each other like a team and condescending tones are not acceptable. So that already is a huge world of difference … And the general etiquette of the ECHO session, how to talk, how to make a presentation, how not to talk down on other people, how not to use insulting terms and all that.’ (*Cancer Focus Group*)
2.d.	Information from didactics	‘And I do like the variety in topics, so as someone who leads LGBTQ+ trainings, I’m in education myself, I can tell you the most frequent request I get is, please don’t give me something general. I’ve listened to so many general talks and trainings, I think you can apply that to any topic in healthcare. So I do think that there are some topics that I haven’t really seen presented elsewhere, which is great.’ (*LGBTQ+ Focus Group*)
2.e.	Being able to present or address difficult cases	‘Honestly, being able to take clients there and present them as a case and get good feedback has made a big change. I’ve been able to bring some of my really complex and weird cases in and get help … ’ (*HIV Interview 1*)
2.f.	Interprofessional nature	‘I like the fact that it is interdisciplinary, which I think is very important for so many reasons. A lot of times when we have these types of rounds or these virtual clinical decision-making things, it tends to be driven a lot by one profession individual, or we tend to get in our specialty silos. So I think the interdisciplinary approach is awesome.’ (*IPM Interview 1*)
2.f.i.	Equity or lack of hierarchy	‘Yeah, I think I like the idea of different people coming together, a multidisciplinary team, in an all-teach, all-learn manner. So, again, it’s not your conventional healthcare system where the consultant is the grand know-it-all.’ (*Cancer Focus Group*)
2.g.	Access to expert opinion	‘At one point, I had questions regarding a surgery, and they were able to bring in a surgeon to address those questions. I mean, it’s just a very, very amazing program. There’s so much value.’ (*LGBTQ+ Interview 2*)
2.h.	Changes to professional practice	‘Well, I would say I’m definitely more knowledgeable in terms of just some of the more up-to-date and multidisciplinary approaches to handling problems.’ (*IPM Interview 2*)
**3.**	**Ways ECHO can improve**	
3.a.	Facilitate networking	‘ … it would be nice that I didn’t have to kind of cold call, send a cold letter to saying, “Hey, I saw you on ECHO.” And kind of reinforce who I am and all this other kind of stuff. There’d be a little bit more that the ECHO could maybe help facilitate that.’ (*HIV Interview 2*)
3.b.	Structural changes	-
3.b.i	Record didactic presentations	‘ … having at least the didactic recorded would be really helpful, you know?’ (*HIV Interview 2*) ‘I know that recordings were not available before, so I’m really happy they are, though. And just because in clinic is really hard to really kind of separate or block that specific timeframe.’ (*HCV Interview 1*)
3.b.ii	Modify didactic presentations	‘Yeah. Maybe have more didactic time in those sessions. Maybe have more … I don’t know, a longer teaching lesson and then still have the case presentations. Maybe have a longer teaching lesson once a quarter.’ (*HCV Focus Group*)
3.b.iii	Allow more time for case management	‘I think for me, maybe it’s because of the structure of the ECHO that I think sometimes it takes a while to get to the good case discussions.’ (*LGBTQ+ Focus Group*)
3.b.iv	Reconceptualize or minimize introductions	‘ … every time I see ECHO, the process of everyone introducing themselves at the beginning of every ECHO goes on for a long time. And I’m 60. I have the capacity to remember two new names on any given day. After that, I’m done. I’m over.’ (*HIV Focus Group*)
3.c.	Expanding the ECHO community	‘If there was a way we could, in between things, have a question submission that you just did online … That might include some things that we aren’t getting covered otherwise … Maybe it could be like Basecamp where you have a platform, and you leave questions and people who have the answers leave the answers. You just get an email notification to check Basecamp, or you can log in and see. It’s a forum.’ (*HCV Focus Group*)
3.d.	Consider braiding telemedicine with ECHO	‘So I wish it was, we were in a room with a patient and there was kind of an active team problem solving approach with that patient. And logistically, that is a very difficult thing to do.’ (*IPM Interview 1*)
3.e.	Work to facilitate follow-up between sessions	‘Okay. It would be nice at the beginning of the session to, and I don’t think they do on any of those, say, “Do you have any questions about previous sessions?” Just a few, five or 10 minutes … ’ (*Cancer Focus Group*)
3.f.	Miscellaneous ways to improve	‘We almost need an ECHO that does nothing but develop resources, and better utilization of resources.’ (*LGBTQ+ Focus Group*)
**4.**	**Barriers to participating**	
4.a.	Time	‘Yeah. It’s hard to schedule out an hour and a half of your day … ’ (*LGBTQ+ Interview 1*)
4.b.	Scheduling	‘Sometimes the timing, but that’s just the nature of the beast, as they say.’ (*Cancer Interview 2*)
4.c.	Miscellaneous barriers	‘For me, because I don’t deal with clinical patients, sometimes the actual clinical case, it’s a little hard for me emotionally, some of these cases. They just seem so difficult, you know? I don’t know if I am saying this right, but it just seems difficult, and it’s just emotionally upsetting a little bit for me.’ (*Cancer Interview 1*)

## Results

### Motivations to join ECHO

#### General continuing education (CE)

Participants most often indicated that they sought out one or more ECHO programs because they wanted to pursue continuing education. They thought that ‘the format was convenient and low stakes’ (HIV Focus Group) and expressed ‘interest … to pick up on ancillary support’ (IPM Focus Group), meaning areas outside of their current specialty.

#### Help for rural/remote providers

Other participants, especially those attending the HIV and HCV programs, noted that issues with access to training and resources were common in rural and remote areas, including low rates of treatment, so ECHO was a convenient way to begin the process of providing access.

#### Intention to develop networking

A few providers also specifically sought out ECHO to build their practice networks (e.g. ‘working with care coordinators and providers from all different corners of the state’ [HIV Focus Group]).

#### Need for CE credits

Others identified free continuing education credits as a motivator to explore ECHO.

### Value of ECHO

#### Networking

Many participants indicated that the networking afforded by ECHO was valuable. For some participants, it was the fact that ‘I can reach out to the ECHO … ’ (LGBTQ+ Interview 2), whereas others saw ECHO as ‘kind of the 21^st^ century version of the old doctor’s lounge’ (HIV Focus Group). In certain cases, the networking was described as going beyond the participant and involving their colleagues who did not attend ECHO. Such non-attenders were described as reaching out to an ECHO attendee to obtain information and the general spread of the content beyond the group itself (‘Even though the person wasn’t on ECHO themselves, those tools are getting shared and spread … ’ [IPM Focus Group]).

#### Met a technical, legal, or CE requirement

Some participants appreciated that CE was available for ‘the different professions each time’ (HIV Focus Group) or that it counted, in some cases, as professional supervision.

#### Structure of ECHO

ECHO Hubs are trained by the ECHO Institute, and so programs have a similar ebb and flow. Some participants ‘like that rhythm and the outline’ (HIV Interview 2) and felt that the whole program setup was ‘well organized [with a] casual and yet professional, friendly milieu’ (LGBTQ+ Interview 2).

#### Information from didactics

Although ECHO is a multifaceted program, participants noted that the lecture components of the program were valuable, and that ‘the variety of presenters and perspectives … [was] very helpful’ (Cancer Interview 1).

#### Being able to present/address difficult cases

Participants expressed that ‘[they] have been able to present some of [their] clients and get really good feedback’ (HIV Interview 1) and that ECHO is great support because ‘for difficult cases’ it can be a ‘hand to hold’ (HCV Interview 1).

#### Interprofessional nature of the program and lack of hierarchy

A substantive portion of the discussion about ECHO’s value revolved around the wide variety of disciplines and perspectives involved. They felt that ‘it’s wonderful because you get interaction with all these other people … not only just the other doctors because [they] all kind of are taught from the same book’ (Cancer Interview 2). This was seen as leveraging ‘different skill sets’ (IPM Interview 2) and enabling practitioners to understand the ‘hurdles [others] face’ (HCV Focus Group). In many cases, these comments extended specifically to the lack of hierarchy in ECHO programs, and that ‘everybody’s comments are valued’ (Cancer Interview 1).

#### Access to expert opinion

Some comments noted the value of having highly skilled participants attend the ECHO or serve as experts. This included both practical outcomes, such as identifying when a client was taking contraindicated medications (HIV Interview 1) and emphasis on best practices: ‘Well, nobody’s talking about 2018. We’re talking about July 2020 … it’s the most up-to-date’ (Cancer Focus Group).

#### Changes to professional practice

Many respondents indicated either that their personal practice had changed or improved or that their overall practice environment had changed because of ECHO, such as ‘doing [their] own case presentations or case conferences’ (HIV Focus Group).

### Ways ECHO can improve

#### Facilitate networking

There was some interest in having ECHO programs facilitate networking by publishing or sharing attendee information with other attendees.

#### Structural changes

There were four different sub-codes related to structural changes that could potentially be made to the ECHO program. These included:

(i) *Record didactic presentations*. There was a high level of interest in accessing recorded didactics for reference. While recordings were always passively available for participants, this feedback led the Hub to directly disseminate recordings via a follow-up communication after each ECHO and select a new cloud service to distribute this resource.

(ii) *Modify Didactic Presentations*. Some participants were interested in longer didactic sessions on occasion, perhaps to ‘go in depth about what we can do to help our clients’ (HIV Interview 1), because ‘even if it’s a very specific topic, 20 minutes [for a didactic session] … is not enough time’ (LGBTQ+ Focus Group).

(iii) *Allow More Time for Case Management*. Conversely, other participants felt that ‘cases get cut off at the end because the didactics can sometimes go … over’ (HIV Interview 1) and hoped for more time, while acknowledging that ‘everyone’s busy … ’ (HCV Focus Group).

(iv) *Reconceptualize or Minimize Introductions*. A few participants wanted the introductions to be configured to take less time. Jokingly, one person noted, ‘as we added more and more people, by the time we get done with introductions, [the ECHO] is going to be over’ (LGBTQ+ Interview 2).

#### Expanding the ECHO community

We observed a high volume of conversation about ways to extend ECHO outside of the sessions themselves. Typically, this included shared workspaces or message boards, but also broader concepts like ‘local ECHOs’ paired with ‘larger ECHOs’ (LGBTQ+ Focus Group).

#### Consider braiding telemedicine with ECHO

Although it was generally seen as difficult to achieve, some participants wanted to see fewer barriers and distance between patients and the ECHO itself, including live, digital case management.

#### Work actively to facilitate follow-up

A few participants were interested in more intentional follow-up on both case management and on understanding from the didactic sessions.

### Barriers to participating

#### Time

Although some participants elsewhere indicated that certain components could be longer, many participants found it difficult to locate 60 or 90 minutes during a workday, especially when they must justify the use of time to an employer (HCV Interview 1).

#### Scheduling

Many participants indicated barriers related to the time of day or day of the week the programs were offered.

## Discussion

Collecting and analyzing data from five different ECHO programs produced a coherent and saturated examination of the ECHO model, leading further toward the desired ‘clear understanding’ and enumeration of ‘necessary components’[18] for study. We note several implications for future research and ideas that ECHO programs might consider exploring, though these do not exhaustively reflect the Results.

(1) Researchers should study one or more outcome measures directly related to the formation of a professional network of practice and learning as an important end in itself. This might also include measures related to job satisfaction or feelings of isolation.

Many practitioners joined and valued ECHO because of the networking that it offered; this was not just a matter of accessing expert opinion but was seen, sometimes, as a replacement for traditional gathering places and a means to reduce practitioner isolation. This finding mirrors qualitative ECHO studies emphasizing community of practice and practitioner isolation [31–35]. It also reflects prior work on andragogical spaces for medical professional identity[36]. Not only did study participants indicate the value of networking, they suggested different ways that ECHO could further facilitate such, or build a community of practice and learning.

(2) Randomized trials of andragogical and patient-level outcomes are warranted, and the scope of outcomes in such studies potentially should be broadened.

Consistent with prior work, our findings suggest that ECHO participants are motivated by and receive quality continuing education [31,32,34,35,37,38] and report changes to their own practice [31,33,35,38]. Indeed, these outcomes often have been foci of quantitative studies collected by systematic reviews [13,15], but the strength of evidence should be increased. Further, our data suggest that both learning and practice change may extend beyond attendees to second- and third-degree contacts. This has been noted previously, but infrequently, in other qualitative work [32–34]. Studies exclusively focused on attendees and their patients/clients may underestimate ECHO’s aggregated impact on outcomes.

(3) ECHO Hubs should consider measuring perceptions of interprofessional collaboration and education (IPC/IPE) when appropriate.

Some ECHO programs clearly offer IPE [39–41] and have explored benefits of IPE[40]. Our study, somewhat uniquely, identified wide-ranging support for the interprofessional components of ECHO, not only for the purposes of IPE but also as a means of leveling practitioner hierarchy in the healthcare education space. Use of a standardized IPE tool [42] to measure participant perceptions may prove informative.

(4) Consider and experiment with ways that barriers to access can be overcome without diluting the model.

Unsurprisingly [32,34,37], time commitment and scheduling were identified as barriers to participating in ECHO; however, the programs in this study were offered for different lengths (60/90 minutes) and on different days and times of day, lending credence to one participant’s comment that it may just ‘be the nature of the beast’ (Cancer Interview 2). Multiple amendments to ECHO were suggested to address this, including recording didactics and minimizing time spent on introductions. However, no granular assessment of the ‘necessary’ components of the ECHO model exists, so the degree to which altering core elements of the model affects outcomes must be studied.

## Strengths and limitations

This study combined qualitative data from five different ECHO programs, thereby reducing the impact of any single program’s idiosyncratic experience on the results. In addition, given the large amount of published ECHO-based research focused on the first four levels of Moore’s framework, this study explicitly was designed to be generative – suggesting new trajectories – rather than focused on learning outcomes and practice change, per se.

However, these data should not, in and of themselves, drive any specific programmatic decisions. Since active ECHO participants were purposively sampled, these data are less likely to reflect perspectives of infrequent attenders. At the same time, there is some preliminary evidence that providers who attend ECHO programs infrequently may primarily be limited by scheduling difficulties[43]. It is also possible that the authors’ own biases unduly affected the results; this risk was reduced by not utilizing the interviewer as a coder or analyst. The incorporation of different ECHO programs meant a wider diversity in perspectives about ECHO as a model, but some broad concepts (e.g. IPE) may be less relevant to an ECHO program focused tightly on a single topic or profession.

## Conclusions

Most research on Project ECHO has focused on identifying participant-level or, more rarely, patient-level outcomes from Moore’s evaluation framework. This study responds to calls to better understand ECHO by analyzing standardized qualitative data from five different ECHO programs with a focus on understanding the ECHO model itself. We suggest four new or modified areas for future research and exploration of this promising andragogical approach.

## Supplementary Material

Supplemental Material

## Data Availability

Please contact the authors regarding availability of the raw data in text format only. Sharing any transcript will be possible pending complete de-identification on behalf of both the participants and the interviewer, as well as removing other details. For reasons of confidentiality, video recordings of interviews and focus groups will not be made available.
