# Supplementary material for: Reflections on project ECHO: qualitative findings from five different ECHO programs
Source: Med Educ Online. 2021 Jun 2;26(1):1936435. doi: 10.1080/10872981.2021.1936435 (PMC8174483; doi:10.1080/10872981.2021.1936435)
Supplement: Supplemental Material [file ZMEO_A_1936435_SM5695.docx]

Guiding Questions for Zoom ECHO Focus Groups 2020

1. Introductions
2. What are your overall impressions of ECHO?
3. What do you like about the ECHO program/sessions?
4. What do you not like about the ECHO program/sessions and how would you change it?
5. In what ways, if any, has your practice changed since participating in the ECHO program?
6. In an ideal world, with no barriers, what would the ECHO program look like?
7. What is your experience with practicing in an interdisciplinary approach?**
8. What is your experience with accessing recordings or information available from past ECHO sessions in box?**
9. How important are the CME/CEU offerings to you?**
10. Do you have any other observations or comments?

**These items (gray text) were not included in the interview guide until after the first 9 data collection events (3 focus groups and 6 interviews). These were areas identified in the initial interviews and focus groups where the Hub wanted to be sure that saturation was reached.
